# Reduced Energy Barrier for Li^+^ Transport Across Grain Boundaries with Amorphous Domains in LLZO Thin Films

**DOI:** 10.1186/s11671-020-03378-x

**Published:** 2020-07-25

**Authors:** Yanlin Zhu, Shuai Wu, Yilan Pan, Xiaokun Zhang, Zongkai Yan, Yong Xiang

**Affiliations:** 1 School of Materials and Energy, University of Electronic Science and Technology of China, Chengdu, 611731 Sichuan China; 2 Advanced Energy Research Institute, University of Electronic Science and Technology of China, Chengdu, 611731 Sichuan China

**Keywords:** Solid electrolytes, LLZO, Thin film, Energy barrier, Ionic conductivity

## Abstract

The high-resistive grain boundaries are the bottleneck for Li^+^ transport in Li_7_La_3_Zr_2_O_12_ (LLZO) solid electrolytes. Herein, high-conductive LLZO thin films with cubic phase and amorphous domains between crystalline grains are prepared, via annealing the repetitive LLZO/Li_2_CO_3_/Ga_2_O_3_ multi-nanolayers at 600 °C for 2 h. The amorphous domains may provide additional vacant sites for Li^+^, and thus relax the accumulation of Li^+^ at grain boundaries. The significantly improved ionic conductivity across grain boundaries demonstrates that the high energy barrier for Li^+^ migration caused by space charge layer is effectively reduced. Benefiting from the Li^+^ transport paths with low energy barriers, the presented LLZO thin film exhibits a cutting-edge value of ionic conductivity as high as 6.36 × 10^−4^ S/cm, which is promising for applications in thin film lithium batteries.

## Introduction

As the rise of 5G mobile telecommunication network, the power consumption of mobile terminals is expected to significantly increase [1–3]. Thin film lithium batteries (TFLBs) with high energy density, long cycle life, and excellent safety hold great promise for the integrated power sources in the intelligent terminals, such as smart cards [4]. To date, most of the workable TFLBs are based on LiPON solid electrolyte [5]. But the low ionic conductivity of LiPON limits the performance of TFLBs. Garnet Li_7_La_3_Zr_2_O_12_ (LLZO) is another promising alternative, due to its high ionic conductivity, wide electrochemical window, and stability against to Li metal anodes [6–10]. However, it remains a challenge to fabricate LLZO thin films with high ionic conductivity [11, 12].

**Fig. 1:**
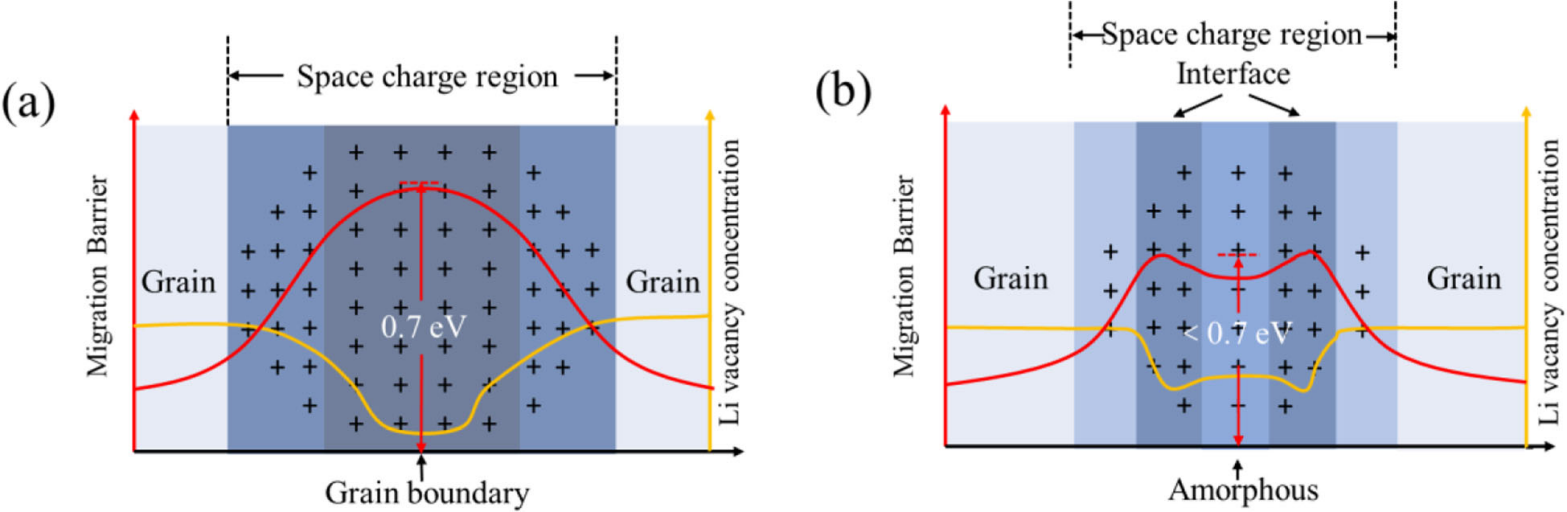
Illustration of the migration barrier and Li vacancy concentration at the conventional grain boundary (**a**), and the grain boundary with amorphous domains (**b**)

It is well-known that the energetically favorable paths for Li^+^ transport are one of the keys to achieving high ionic conductivity in solids [13, 14]. For the case of polycrystalline LLZO thin films, there are two energy barriers that determine the Li^+^ conducting performance. One is related to Li^+^ transport within a grain. The lattice sites possibly occupied by Li^+^ are energetically nonequivalent, and thus Li^+^ must get over an energy barrier (EB_g_) when it hops between these sites [15–18]. The other one is related to Li^+^ transport across the grain boundaries (GBs) [19, 20]. The lattice defects at GBs would cause the accumulation of Li^+^. A space charge layer would form because the unoccupied possible sites for Li^+^ around GBs are depleted (orange line in Fig. 1a). The space charge effect results in a high migration energy barrier (EB_gb_, red line in Fig. 1a) [21]. Typically, EB_gb_ (~ 0.7 eV) is much higher than EB_g_ (~ 0.3 eV) for the case of LLZO [20].

It has been reported that the possible sites for Li^+^ occupation in the LLZO with cubic phase, which are tetrahedral 24d site (Li1) and distorted octahedral 96 h site (Li2), are close to energetically equivalent [16, 22, 23]. Therefore, it is generally believed that the EB_g_ in the cubic LLZO is moderate (~ 0.3 eV). Although the cubic phase of LLZO is metastable at room temperature (RT), the strategies to stabilize it through the doping of high valence cations, such as Al^3+^, Ga^3+^, and Ta^5+^, have been well developed [24–33]. Lobe et al. reported Al-doped LLZO thin films with ionic conductivity of 1.2 × 10^−4^ S/cm and activation energy of 0.47 eV [34]. It is generally believed that the high concentration of Li^+^ in the crystal lattice may further help to lower EB_g_ [11, 13]. LLZO thin films with activation energy of 0.38 ± 0.02 eV have been prepared by introducing extra Li_2_O during thin film deposition [12, 35]. Li_2_O effectively compensated the lithium loss during sputtering-deposition. On the other hand, the strategy to address the conduction issues derived from high EB_gb_ is few, although it is well-known the high-resistive GBs is the bottleneck for Li^+^ transport in LLZO [14, 21].

**Fig. 2:**
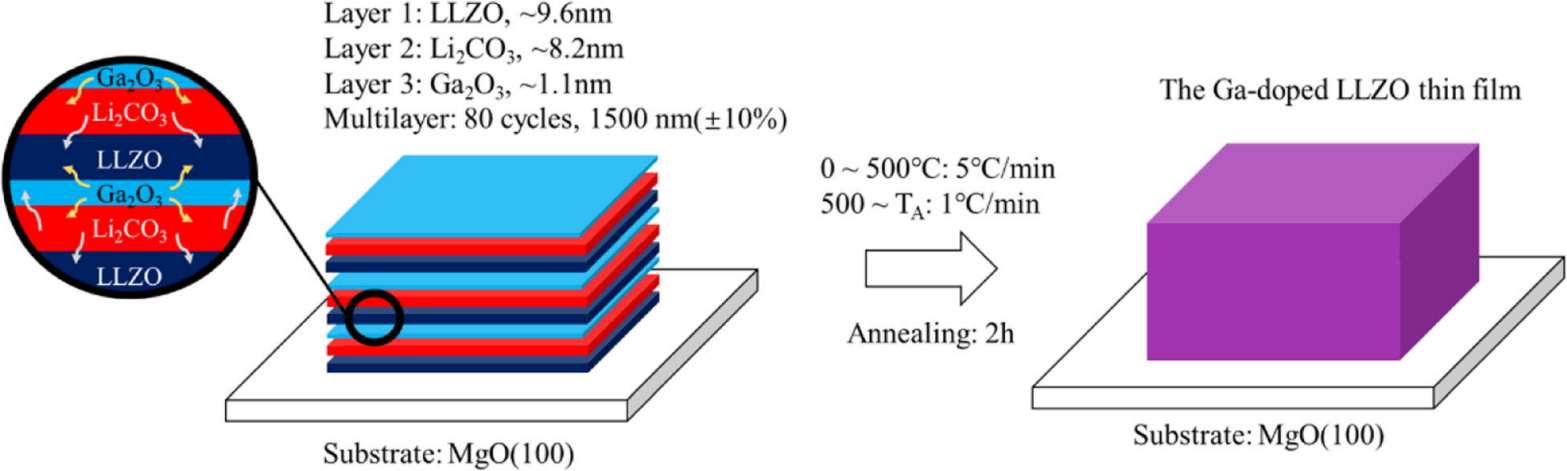
Schematics of fabrication procedures of the presented LLZO thin films

In this work, we demonstrate a LLZO thin film with amorphous domains between crystalline grains. The amorphous domains could provide extra Li^+^ vacancies [21, 36–38] and a lower migration barrier (~ 0.6 eV) [36] at GBs (Fig. 1b), which would weaken the space charge effect and lower EB_gb_ (< 0.7 eV) [21, 38]. The presented LLZO thin film is prepared via repeatedly depositing the sequentially stacked nanolayers of LLZO, Li_2_CO_3_, and Ga_2_O_3_, and the following annealing (Fig. 2). The ultrathin thicknesses of each layer facilitate the interdiffusion in the multilayered structure, in turn enable Ga_2_O_3_ to help to stabilize the cubic phase of LLZO, and Li_2_CO_3_ to compensate the Li loss during deposition and annealing. Through carefully tuning the temperature of annealing, the LLZO thin film with the desired cubic phase and amorphous domains between grains was obtained. The electrochemical impedance measurement suggests the presented LLZO thin film solid electrolyte achieves a high ionic conductivity of 6.36 × 10^−4^ S/cm.

## Methods

### Fabrication of Ga-LLZO Thin Film Solid-State Electrolyte

The ultrathin layers of LLZO, Li_2_CO_3_, and Ga_2_O_3_ were sequentially deposited by radio frequency magnetron sputtering on polished MgO (100) substrates in pure Ar atmosphere. A multilayered thin film with the thickness of ~ 1500 nm (± 10%) was obtained by repeatedly deposited the triple-layered unit for 80 cycles (Figure S1). The targets of Li_7_La_3_Zr_2_O_12_ (99%), Li_2_CO_3_(99%), and Ga_2_O_3_(99.9%) mounted on 190 mm × 55 mm Cu backing plates are provided by Zhongnuo New Materials Manufacturing Co., China. The LLZO target used here is with desired cubic phase (Figure S2) and its density is 5.35 g/cm^3^. The pressure for the deposition is 1 Pa. The power density for LLZO deposition was 2.38 W cm^−2^, and 1.90 W cm^−2^ for Li_2_CO_3_ and Ga_2_O_3_. The as-deposited multilayered thin films were further annealed in pure oxygen (99.99%) for 2 h at 600 °C, 700 °C, and 800 °C, respectively.

### Characterization

The thickness of each single layer of LLZO, Li_2_CO_3_, and Ga_2_O_3_ was determined by a step profiler (see details in Note S1 and Table S1). The crystallographic structure of the thin film was determined using X-ray diffraction (XRD), with Cu-Kα source and 2θ in the range from 10 to 60°. The chemical composition was characterized using time-of-flight secondary ion mass spectrometry (TOF-SIMS) and high-resolution transmission electron microscopy (HRTEM) equipped with an energy dispersive X-ray spectroscopy (EDX) detector. The ionic conductivity was determined in an in-plane test configuration at room temperature (25 °C), via measuring electrochemical impendence spectroscopy (EIS) with the applied frequency ranged from 3 × 10^6^ to 1 Hz with a constant 30 mV AC amplitude. The aluminum contacts on the top of LLZO thin films were fabricated using direct current magnetron sputtering. The data of EIS was processed using the Zview software.

## Results and Discussion

**Fig. 3:**
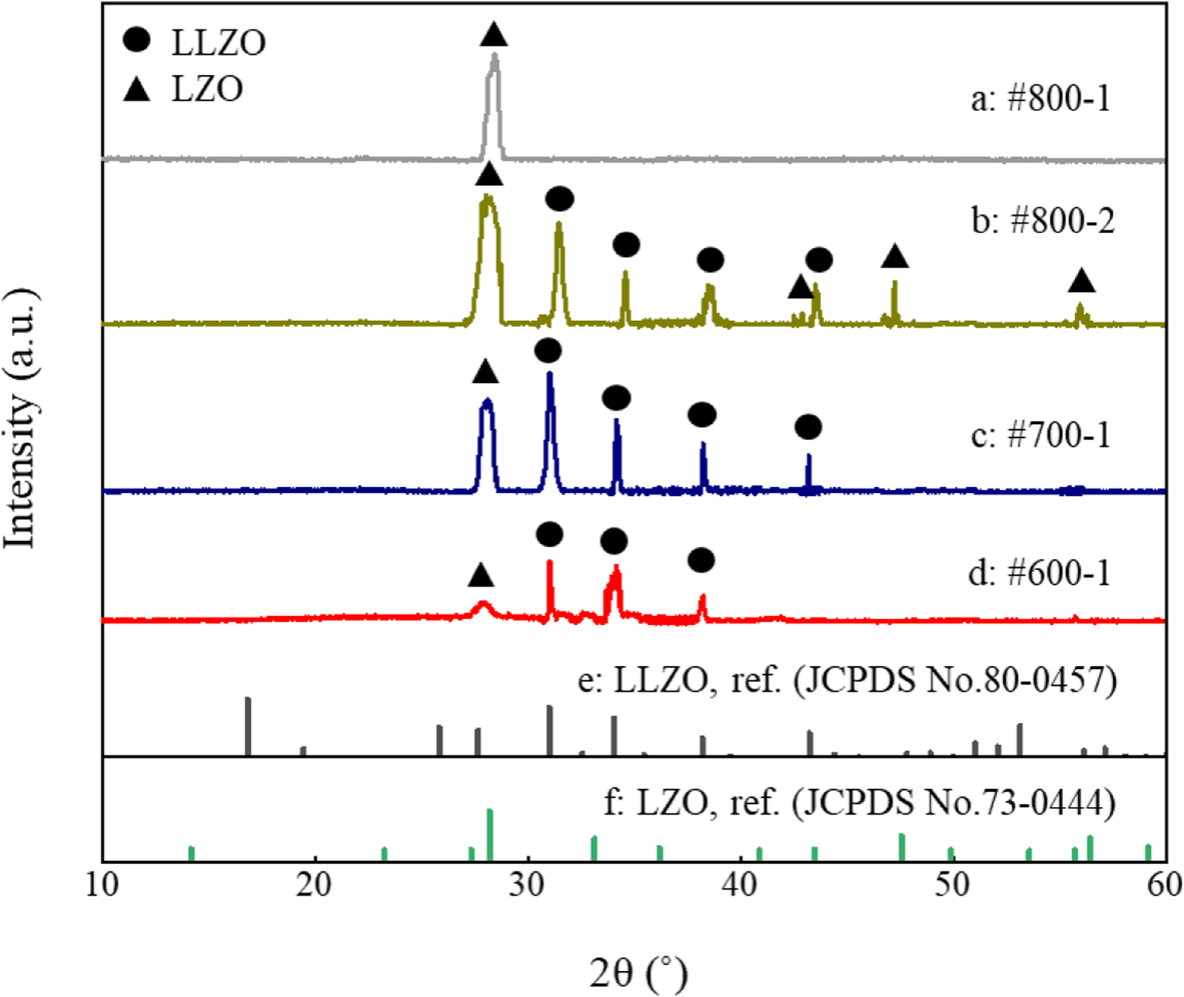
XRD patterns of #800-1 (**a**), #800-2 (**b**), 700-1 (**c**), and #600-1 (**d**), and the standard diffraction patterns for cubic LLZO (**e**) and LZO (**f**)

The LLZO thin film samples and their process parameters were summarized in Table 1. Sample #800-1 without Li-supplementary and Ga-doping exhibits a Li-deficient phase of La_2_Zr_2_O_7_ (LZO) after annealing at 800 °C for 2 h (Fig. 3a). After introducing Ga_2_O_3_ and Li_2_CO_3_, the diffraction peaks belonging to the cubic phase of LLZO are observed in the XRD pattern of #800-2 (Fig. 3b). This suggests that Ga dopant and extra Li would be favorable for the formation and/or stabilization of the desired cubic phase of LLZO. However, a strong diffraction peak at 28.2° indexed to LZO remains in the XRD pattern of #800-2. As the annealing temperature decreases to 700 °C, the intensity of the diffraction peak at 28.2° declined appreciably (Fig. 3c). These observations indicate that the high temperature annealing may lead to a severe Li loss even though extra Li is introduced. Through further reducing the annealing temperature to 600 °C, the thin film with a major phase of cubic LLZO and a negligible diffraction peak of LZO were obtained (Fig. 3d). Our observations are consistent with previous literature [11, 12], which report that the formation of the cubic phase in Ga-doped LLZO thin films is triggered at 600 °C, and LZO may form within 700 to 800 °C.

**Table 1:** Samples of LLZO thin film solid electrolyte and their preparation parameters

Samples	Annealing temperature (°C)	Gallium doping	Extra lithium
#800-1	800	×	×
#800-2	800	√	√
#700-1	700	√	√
#600-1	600	√	√

**Fig. 4:**
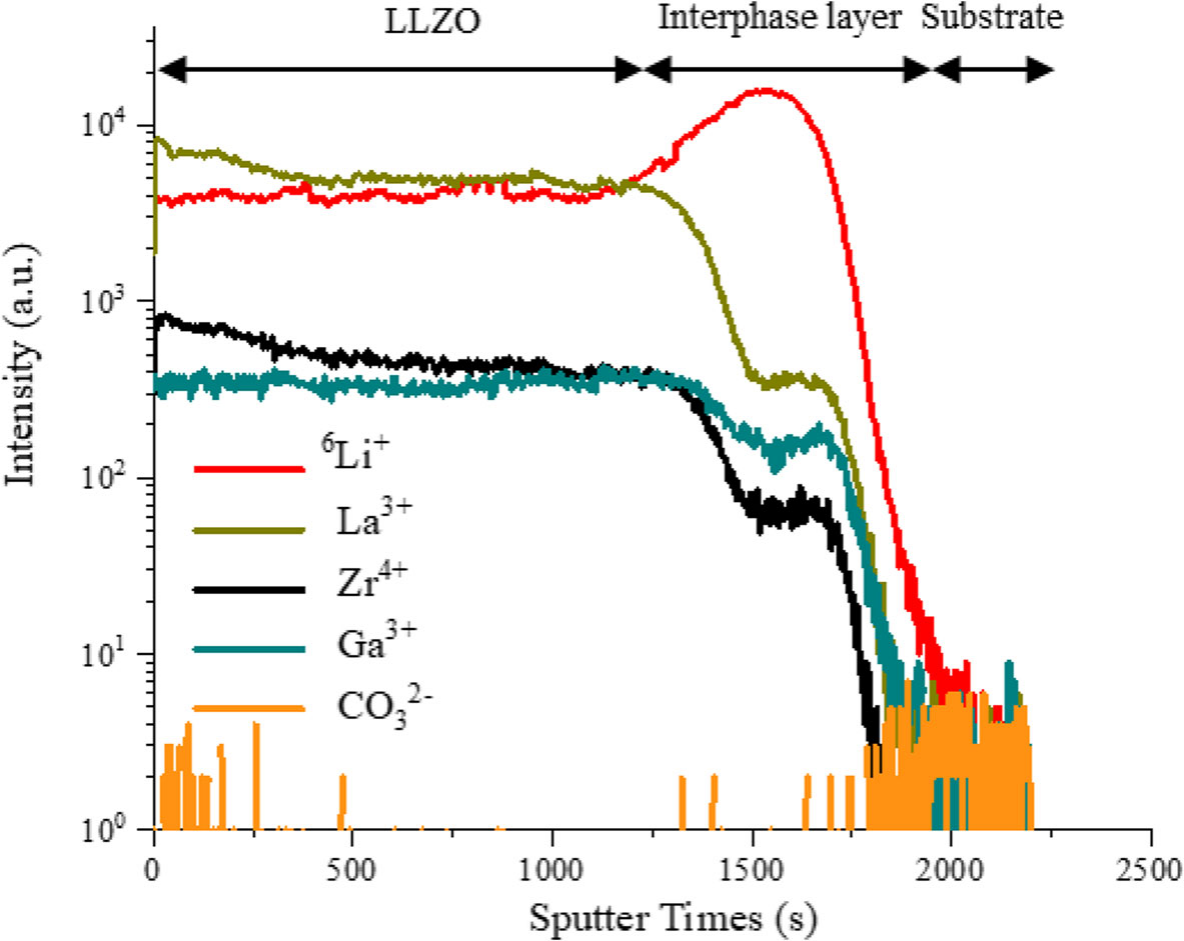
TOF-SIMS depth profiles of #600-1: ^6^Li^+^ (red), La^3+^ (green), Zr^4+^ (black), Ga^3+^ (indigo), CO_3_^2−^ (orange)

Meanwhile, there are no diffraction peaks of Li_2_CO_3_ or Ga_2_O_3_ observed in the XRD patterns (Fig. 3). In addition, the compositional depth profile of #600-1 obtained using TOF-SIMS shows that the signal of CO_3_^2−^ is very low through the whole thin film (orange line in Fig. 4). And the competent content of Li in #600-1 is demonstrated by the high intensity of the recorded counts of ^6^Li^+^ (red line in Fig. 4). Thus, Li_2_CO_3_ in the multilayered thin film should have completely decomposed after annealing at 600 °C for 2 h, and effectively compensated the Li loss during thin film deposition and heat treatment. In addition, the undesired reaction between LLZO and CO_2_, which may form a low-conductive layer of Li_2_CO_3_, should be effectively prevented by the annealing atmosphere of pure oxygen. This inference is consistent with the measured high ionic conductivity of #600-1 (see below).

TOF-SIMS characterization also reveals the even distribution of ^6^Li^+^, La^3+^, Zr^4+^, and Ga^3+^ throughout the thin film #600-1 (Fig. 4). Typically, the interdiffusion of the precursors should be the speed control step in solid-state reactions. Huang et al. reported that the interdiffusion distance of the Ga_2_O_3_ and LLZO precursor layers was about 10–20 nm during an annealing process at 700 to 900 °C for 2 h. Thus, the thickness of each precursor layer in this study was set to be less than 10 nm. The multilayered structure based on the nanolayers of LLZO, Li_2_CO_3_, and Ga_2_O_3_ fabricated here, facilitates the homogenous mixing of the precursors via reducing their necessary diffusion length significantly. The uneven distribution of doped element observed in the LLZO thin films derived from the thicker precursor layers [11] are not observed here. An enrichment of Li in the interphase layer between the deposited thin film and MgO substrate can be observed. This should ascribe to the diffusion of Li^+^ into MgO lattice [34].

Briefly, the multilayers of LLZO/Li_2_CO_3_/Ga_2_O_3_ are well-mixed and reacted, benefiting from the sufficient interdiffusion among these ultrathin layers. Moreover, the reaction kinetics in the multilayered thin films with doped Ga and extra Li are optimized at 600 °C, for the sake of trying to prepare the cubic phase of LLZO with a low EB_g_.

**Fig. 5:**
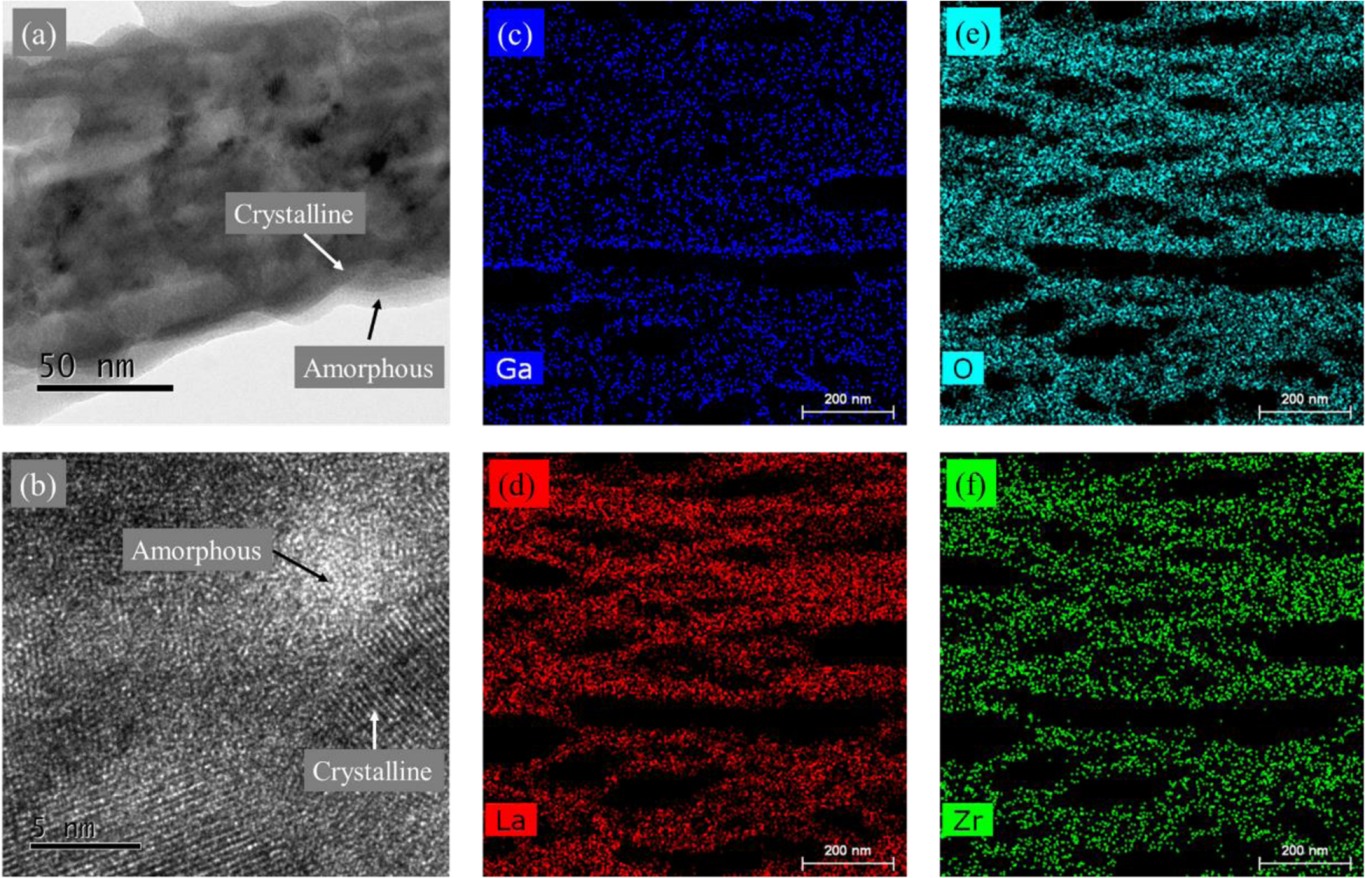
HRTEM images (**a**, **b**) and elemental mapping (**c** for Ga, **d** for La, **e** for O, **f** for Zr) of LLZO thin film #600-1

As mentioned above, the Li^+^ conducting performance of LLZO is notably influenced by the structures at GBs (Fig. 1). The microstructure of #600-1 is carefully characterized using HRTEM. The crossed structure, which is a typical indicator of the reactions between LLZO and H_2_O or CO_2_ [35], can be observed in the HRTEM images. However, the XRD pattern and TOF-SIMS depth profile of #600-1 suggest that the as-prepared LLZO thin films prevent from reacting with H_2_O or CO_2_. Thus, it is reasonable to ascribe the formation of crossed structure to the exposure of LLZO thin films to air during the preparation of testing samples. Remarkably, amorphous domains between crystalline grains are observed (Fig. 5a, b). It indicates that #600-1 LLZO thin film should be not fully crystallized after annealing, which is consistent with the relative large full width at half maximum (FWHM) observed in the XRD pattern of #600-1 (Fig. 3d). EDX mapping reveals the uniform distribution of Ga, La, O, and Zr over the crystalline grains and amorphous domains (Fig. 5c–f). Therefore, we propose that the amorphous domains are composed of glassy Li-Ga-La-Zr-O oxides. It has been known that amorphous LLZO is a Li^+^ conductor. Its typical ionic conductivity and activation energy are 1 × 10^−6^ S/cm and ~ 0.6 eV, respectively [36]. The Li^+^-conductive amorphous domains would improve the physical contact between crystalline grains, and thus, the paths for Li^+^ transport in the thin films are with a better continuity [20]. More importantly, the amorphous domains between the grains are potential to provide additional vacant sites for Li^+^ [21, 36–38]. The electrostatic repulsion between Li^+^ would be reduced, compared with the conventional LLZO GBs in which the possible sites for Li^+^ occupation are depleted [19, 20]. In other words, the amorphous domains may diminish the cacoethic space charge effects and lower the EB_gb_ for Li^+^ transport across GBs (Fig. 1b). Consequently, it is reasonable to expect a reduced grain boundary resistance (R_gb_) in the present LLZO thin film solid electrolyte #600-1.

**Fig. 6:**
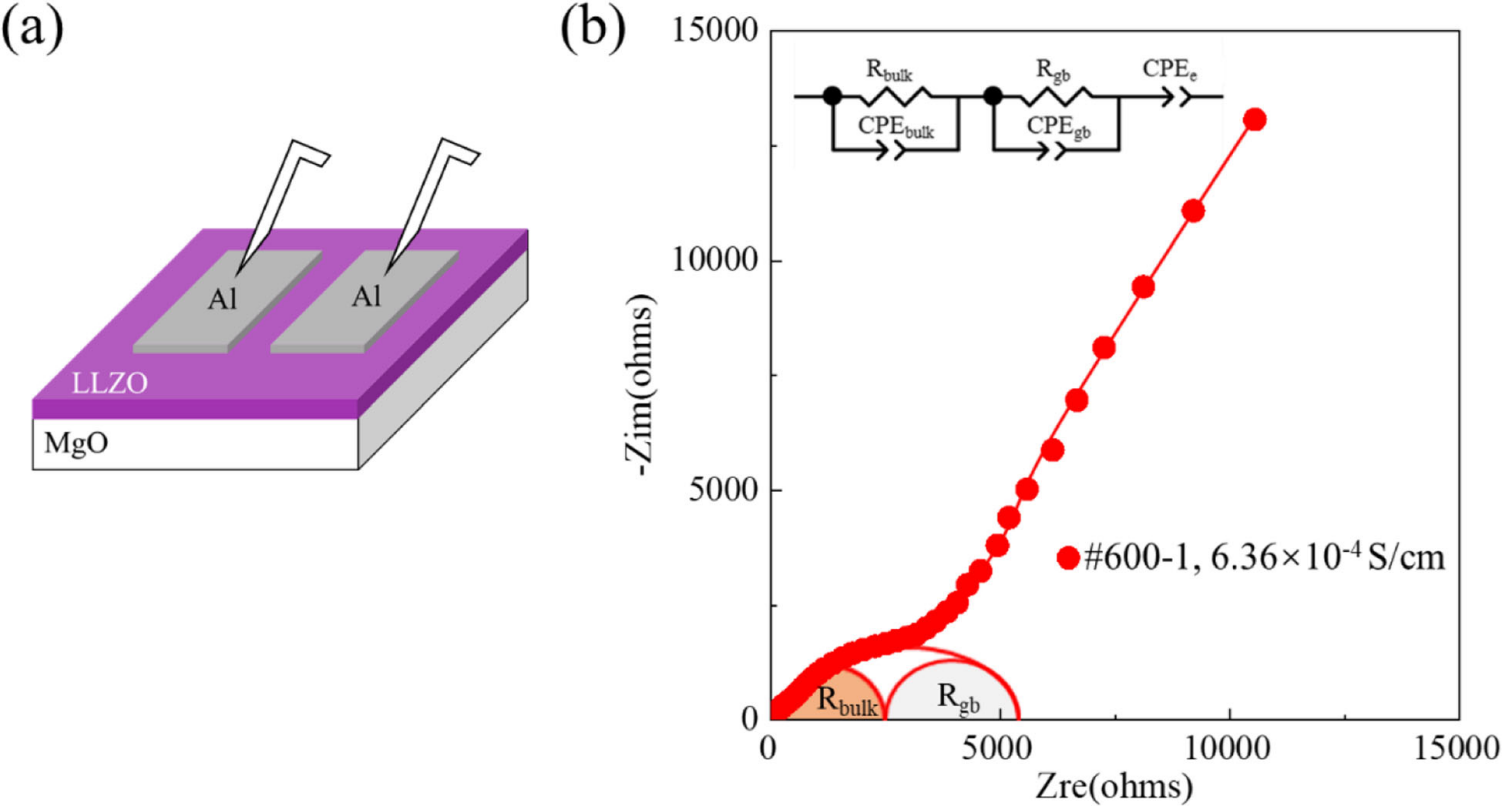
**a** The in-plane test configuration for EIS measurements. **b** The Nyquist plot of impedance spectrum of LLZO thin film #600-1 measured at room temperature, insert shows the equivalent circuit for EIS analysis

The EIS measurements of the presented LLZO thin films are conducted with the in-plane test configuration shown in Fig. 6a. Their total ionic conductivities (*σ*_total_) can be calculated according to the equation:

1 $$ {\sigma}_{\mathrm{total}}=\frac{L}{\mathrm{S}{\mathrm{R}}_{\mathrm{total}}} $$

where *L* is the distance between the two contacting electrodes, *S* is the electrode area, and *R*_total_ is the total resistance of LLZO thin film determined through EIS measurements. The Nyquist plots of the measured impedance spectra (Fig. 6b and Figure S2a and S2b) are fitted with the equivalent circuit depicted in the inserts, which consists of a series combination of a constant phase element (CPE) with two circles of a resistor in parallel with a CPE. *R*_bulk_ and *R*_gb_ in the equivalent circuit represent the bulk resistance and the grain boundary resistance of the LLZO thin film. The grain boundary ionic conductivities (*σ*_gb_) of LLZO thin films are also normalized to the distance of two parallel contacting electrodes, and can be calculated according to the following equation [39]:

2 $$ {\sigma}_{\mathrm{gb}}=\frac{L}{\mathrm{S}{\mathrm{R}}_{\mathrm{gb}}}\frac{C_{\mathrm{bulk}}}{C_{\mathrm{gb}}} $$

where *C*_bulk_ and *C*_gb_ are the bulk capacitance and the grain boundary capacitance, which can be calculated using the equation (3) based on the fitted values of their corresponding *R* (*R*_bulk_ and *R*_gb_) and CPE (CPE_bulk_ and CPE_gb_) [34, 40].

3 $$ C={\left(\mathrm{CPE}\times {R}^{1-\mathrm{n}}\right)}^{\frac{1}{\mathrm{n}}} $$

The geometrical parameters (*L* and *S*) and the fitted values of the elements in the equivalent circuit (*R*_total_, *R*_bulk,_*R*_gb_, *C*_bulk_, and *C*_gb_) are summarized in Table S2. Table 2 summarizes the calculated *σ*_bulk_, *σ*_gb_, and *σ*_total_ at room temperature of the presented LLZO thin films. *σ*_total_ of #800-1 is lower than 10^−8^ S/cm since it is dominated by the Li-poor phase of LZO. The samples with Ga dopant and extra Li, #800-2, #700-1, and #600-1, possess the *σ*_total_ of 5.63 × 10^−7^, 3.89 × 10^−5^, and 6.36 × 10^−4^ S/cm, respectively. This trend may be caused by two reasons. First, the proportion of high-resistive LZO in the prepared thin films is trimmed down as the annealing temperature is reduced, which is demonstrated by their XRD patterns (Fig. 3b–d). Second, the intensities of the diffraction peaks of #600-1 are much lower than that of the other two. Its low crystallinity may be related to the formation of amorphous domains between crystalline grains. As mentioned above, the amorphous domains between crystalline grains may lower the energy barrier for Li^+^ transport across GBs (Fig. 1). In addition, the grain size of #600-1 is about 50 nm (Figure S3), which is smaller than the common values (hundreds of nanometers) reported in previous studies and may lead to a greater number of high-resistive GBs. However, the ionic conductivity of #600-1 reaches a cutting-edge value. These facts give a good indication that the strategy presented here to lower the energy barrier for Li^+^ transport across GBs is effective. The analysis of EIS data indeed shows that *σ*_gb_ of #600-1 is closed to 2 orders of magnitude higher than that of #700-1, although it is difficult to quantify *σ*_bulk_ and *σ*_gb_ of #800-1 and #800-2 because of their high grain boundary resistance.

**Table 2:** Bulk ionic conductivities (*σ*_bulk_), grain boundary ionic conductivities (*σ*_gb_), and total ionic conductivities (*σ*_total_) at room temperature of the presented LLZO thin films

Sample name	*σ*_bulk_ (S/cm)	*σ*_gb_ (S/cm)	*σ*_total_ (S/cm)
#800-1	/	/	7.86 × 10^−9^
#800-2	/	/	5.63 × 10^−7^
#700-1	1.03 × 10^−4^	6.23 × 10^−6^	3.89 × 10^−5^
#600-1	1.33 × 10^−3^	1.21 × 10^−4^	6.36 × 10^−4^

## Conclusions

In summary, LLZO thin films with cubic phase and amorphous domains between crystalline grains were obtained through introducing Ga dopant and extra Li, and carefully optimizing annealing temperature. Firstly, the small energy disparity between Li^+^ sites in the LLZO lattice of the cubic phase leads to a low energy barrier for Li^+^ transport within crystalline grains. More importantly, the amorphous domains provide additional Li^+^ vacant sites around GBs and thus lower the energy barriers for Li^+^ transport across GBs via relaxing the space charge effects. As a result, benefiting from the Li^+^ transport paths with low migration energy barriers, the presented LLZO thin film exhibits an ionic conductivity of 6.36 × 10^−4^ S/cm at room temperature, which is attractive for applications in TFLBs.

## Supplementary information

**Additional file 1: **Reduced energy barrier for Li^+^ transport across grain boundaries with amorphous domains in LLZO thin-films. **Note S1:** The determination of thicknesses of each single layer of LLZO, Li_2_CO_3_, and Ga_2_O_3._**Table S1.** The thickness of each single-layer thin film. **Table S2.** The geometrical parameters (L and S) of electrodes and the fitted values of the elements in the equivalent circuit (R_total_, R_bulk_, R_gb_, C_bulk_, and C_gb_) of the different thin films for calculating σ_total_, σ_bulk_, and σ_gb_ at room temperature. **Figure S1.** The thickness of #600-1 (1.516 μm) determined in its cross-sectional SEM image. **Figure S2.** XRD patterns of the LLZO target used in this study. **Figure S3.** The grain size of #600-1(~50 nm) determined by SEM image. **Figure S4.** The Nyquist plots of impedance spectra of LLZO thin-films #700-1 (a), #800-1 (green in b), and #800-2 (brown in b) measured at room temperature, inserts show the equivalent circuits for EIS analysis.

## Data Availability

The authors declare that the materials and data are promptly available to readers without undue qualifications for material transfer agreements. All data generated or analyzed during this study are included in this article.

## Abbreviations

Li: Lithium
Li_7_La_3_Zr_2_O_12_ (LLZO): Lithium lanthanum zirconate
La_2_Zr_2_O_7_ (LZO): Lanthanum zirconate
Li_2_CO_3_: Lithium carbonate
Ga_2_O_3_: Gallium(III) oxide
MgO: Magnesium Oxide
Ga: Gallium
La: Lanthanum
O: Oxygen
Zr: Zirconium
Al: Aluminum
Ta: Tantalum
Ar: Argon
Cu: Copper
TFLBs: Thin film lithium batteries
LiPON: Lithium phosphorus oxynitride
Li_2_O: Lithium oxide
EB_g_: Migration energy barrier for Li^+^ transport within a grain
EB_gb_: Migration energy barrier for Li^+^ transport across the grain boundaries
GBs: Grain boundaries
*σ*_total_: Total ionic conductivity
*σ*_gb_: Grain boundary ionic conductivity
*σ*_bulk_: Bulk ionic conductivity
*C*_bulk_: Bulk capacitance
*C*_gb_: Grain boundary capacitance
*R*: Resistor
CPE: Constant phase element
*L*: Distance between the two contacting electrodes
*S*: Electrode area
XRD: X-ray diffraction
TOF-SIMS: Time-of-flight secondary ion mass spectrometry
HRTEM: High-resolution transmission electron microscopy
EDX: Energy dispersive X-ray spectroscopy
EIS: Electrochemical impendence spectroscopy
AC: Alternating current
