# Supplementary material for: Reduced Energy Barrier for Li+ Transport Across Grain Boundaries with Amorphous Domains in LLZO Thin Films
Source: Nanoscale Res Lett. 2020 Jul 25;15:153. doi: 10.1186/s11671-020-03378-x (PMC7382668; doi:10.1186/s11671-020-03378-x)
Supplement: Supplementary file 1 — Additional file 1: Reduced energy barrier for Li+ transport across grain boundaries with amorphous domains in LLZO thin-films. Note S1: The determination of thicknesses of each single layer of LLZO, Li2CO3, and Ga2O3.Table S1. The thickness of each single-layer thin film. Table S2. The geometrical parameters (L and S) of electrodes and the fitted values of the elements in the equivalent circuit (Rtotal, Rbulk, Rgb, Cbulk, and Cgb) of the different thin films for calculating σtotal, σbulk, and σgb at room temperature. Figure S1. The thickness of #600-1 (1.516 μm) determined in its cross-sectional SEM image. Figure S2. XRD patterns of the LLZO target used in this study. Figure S3. The grain size of #600-1(~50 nm) determined by SEM image. Figure S4. The Nyquist plots of impedance spectra of LLZO thin-films #700-1 (a), #800-1 (green in b), and #800-2 (brown in b) measured at room temperature, inserts show the equivalent circuits for EIS analysis. [file 11671_2020_3378_MOESM1_ESM.docx]

Supporting Information for Nanoscale Research Letters

**Reduced energy barrier for Li^+^ transport across grain boundaries with amorphous domains in LLZO thin-films**

Yanlin Zhu^1^, Shuai Wu^1^, Yilan Pan^1^, Xiaokun Zhang^1^*, Zongkai Yan^1^*, Yong Xiang^1,2^*

^1^School of Materials and Energy, University of Electronic Science and Technology of China, Chengdu 611731, Sichuan, China.

^2^Advanced Energy Research Institute, University of Electronic Science and Technology of China, Chengdu 611731, Sichuan, China.

*Correspondence: zxk@uestc.edu.cn (X Zhang), yanzongkai@uestc.edu.cn (Z Yan), xyg@uestc.edu.cn (Y Xiang)

**Note S1: The determination of thicknesses of each single layer of LLZO, Li_2_CO_3_, and Ga_2_O_3_**

The homemade RF magnetron sputtering system used in this study was in a “target-up” configuration with 3 vacuum chambers for different targets. The target with Cu backing plate was mounted on top of the chamber. The substrate could move parallel to the target. The thickness of the deposited thin film was mainly influenced by the moving speed of the substrate and the power density for sputtering. To determine the thickness of each single-layer of LLZO, Li_2_CO_3_, or Ga_2_O_3_, we firstly tested the total thickness (repeatedly deposit the single layer for 200 cycles) of the three materials respectively via step profiler. And the thickness of each single-layer was determined via dividing the measured total thickness by the deposition cycles.

**Table S1**. The thickness of each single-layer thin film.

| **Target** | **Substrate speed (mm/min)** | **Power density**  **(W cm^-2^)** | **Cycles** | **Total thickness**  **(nm)** | **Single-layer thickness**  **(nm)** |
| --- | --- | --- | --- | --- | --- |
| LLZO | 300 | 2.38 | 200 | 1920 | 9.6 |
| Ga_2_O_3_ | 3000 | 1.90 | 200 | 200 | 1.1 |
| Li_2_CO_3_ | 300 | 1.90 | 200 | 1640 | 8.2 |

**
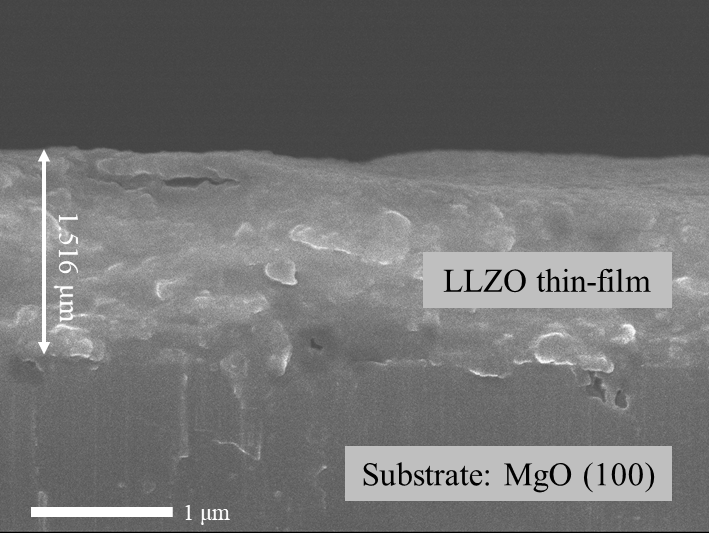
**

**Figure S1.** The thickness of #600-1 (1.516 μm) determined in its cross-sectional SEM image.


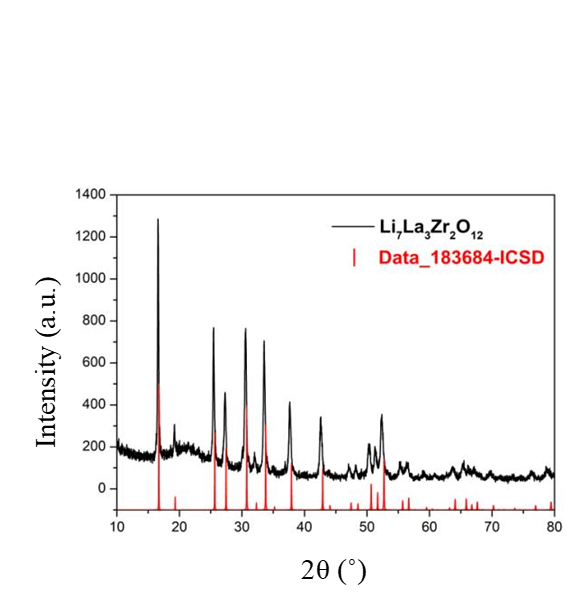


**Figure S2**. XRD patterns of the LLZO target used in this study.


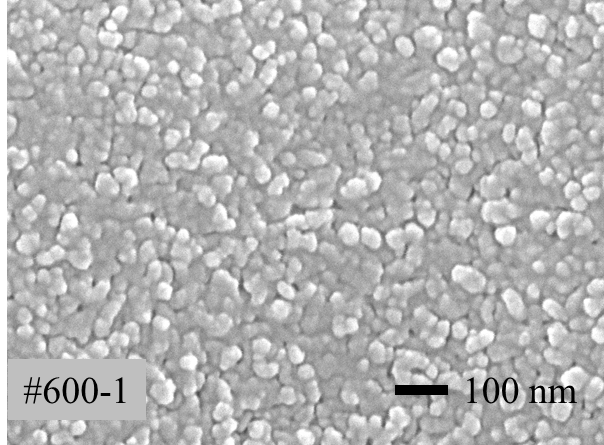


**Figure S3**. The grain size of #600-1(~50 nm) determined by SEM image.


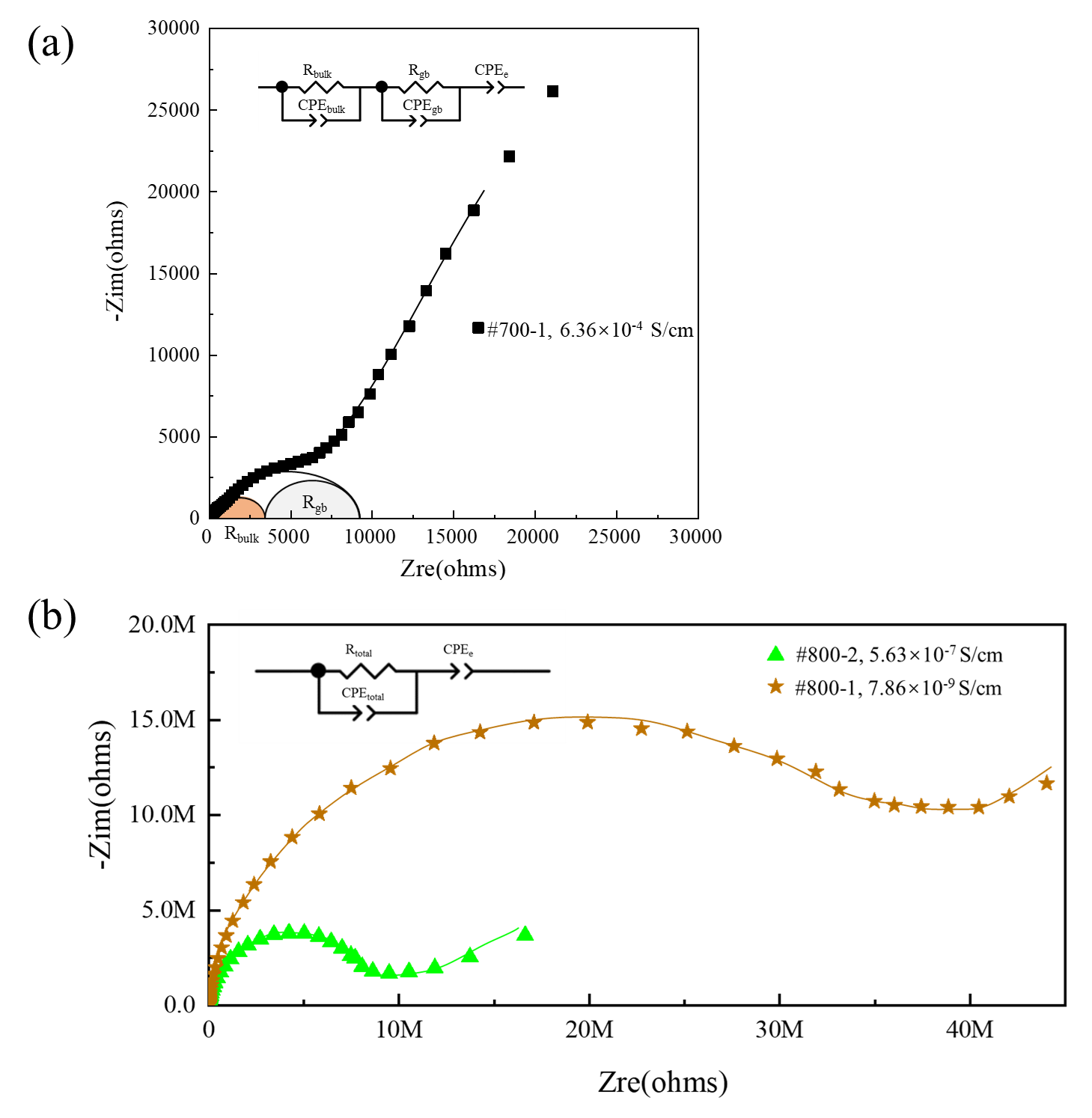


**Figure S4.** The Nyquist plots of impedance spectra of LLZO thin-films #700-1 (a), #800-1 (green in b), and #800-2 (brown in b) measured at room temperature, inserts show the equivalent circuits for EIS analysis.

**Table S2**. The geometrical parameters (L and S) of electrodes and the fitted values of the elements in the equivalent circuit (R_total_, R_bulk_, R_gb_, C_bulk_, and C_gb_) of the different thin films for calculating σ_total_, σ_bulk_, and σ_gb_ at room temperature.

| **Samples** | **L (cm)** | **S (cm^2^)** | **R_total_ (Ω)** | **R_bulk_ (Ω)** | **R_gb_ (Ω)** | **C_bulk_ (F)** | **C_gb_ (F)** |
| --- | --- | --- | --- | --- | --- | --- | --- |
| #800-1 | 0.3 | 1.1372 | 33563278 | / | / | / | / |
| #800-2 | 0.3 | 0.5384 | 9896543 | / | / | / | / |
| #700-1 | 0.3 | 0.9025 | 8545 | 3210 | 5335 | 3.12×10^-10^ | 5.61×10^-10^ |
| #600-1 | 0.3 | 0.0900 | 5241 | 2501 | 2740 | 1.09×10^-10^ | 1.10×10^-9^ |
